# Survival probability and prognostic factors for breast cancer patients in Vietnam

**DOI:** 10.3402/gha.v6i0.18860

**Published:** 2013-01-17

**Authors:** Nguyen H. Lan, Wongsa Laohasiriwong, John F. Stewart

**Affiliations:** 1 Graduate School, Khon Kaen University, Khon Kaen, Thailand; 2 Hue College of Medicine and Pharmacy, Hue University, Hue, Vietnam; 3 Faculty of Public Health, Khon Kaen University, Khon Kaen, Thailand; 4 Department of Economics, University of North Carolina, Chapel Hill, NC, USA

**Keywords:** breast cancer, survival, prognostic factors, Vietnam

## Abstract

**Background:**

Breast cancer is becoming a public health problem in Vietnam. The mortality to incidence ratio of the disease was ranked second among the most common cancers in women. This study estimates the survival probability at 1, 3, and 5 years following diagnosis and determines prognostic factors for breast cancer mortality in Vietnam.

**Methods:**

A survival analysis was conducted based on retrospective data from Hue Central Hospital and the Cancer Registry in Ho Chi Minh City. Using the Kaplan-Meier method, the survival probability of patients with breast cancer was estimated at 1, 3, and 5 years following diagnosis. The covariates among prognostic factors for survival time were studied using an extended Cox proportion hazards model, including time-dependent predictors.

**Results:**

Overall survival rates at 1, 3, and 5 years following diagnosis were 0.94, 0.83 and 0.74 respectively. Marital status, education level, stage at diagnosis, and hormone therapy were prognostic factors for mortality. For the stage at diagnosis, the relation to the risk of death for breast cancer was 1.32 (95% CI, 1.22–1.41). Married women faced a risk of death nearly 1.59 times higher than unmarried women (95% CI, 1.09–2.33). Women with higher levels of education and who received hormone therapy had approximately 10% (hazard ratio [HR]: 0.92; 95% CI, 0.89–0.96) and 80% (HR: 0.22; 95% CI, 0.12–0.41) risk reduction of death respectively, compared with those classified as illiterate and those without hormone therapy.

**Conclusions:**

The 5-year survival probability of breast cancer was lower in Vietnam than in countries with similar distributions of the stage at diagnosis. Screening programs and related support policies should be developed to increase the life expectancy of women with breast cancer in Vietnam.

Breast cancer is the most common cancer among women, with more than 1.1 million women newly diagnosed globally every year. Death from breast cancer accounts for 1.6% of female deaths every year worldwide (1). The mortality rates of breast cancer vary among different communities and countries. The 5-year overall survival rate tends to be lower in developing countries (2, 3). The relationship between survival time and stage at diagnosis is consistent in the literature (4–7). Detection at an earlier stage of the disease and better access to effective treatment have been recommended as solutions to improve the life expectancy of breast cancer patients (3, 8). Other prognostic factors, such as demography, socioeconomics, hormone receptors, and psychology, were found to have varying effects on breast cancer survival rates in different studies (9–14).

The breast cancer incidence in Vietnam has increased steadily over the last decade from a crude rate of 13.8 per 100,000 women in 2000 to 28.1 per 100,000 women in 2010, with an estimated 12,533 breast cancer cases in the country (15). The mortality to incidence ratio of breast cancer was 0.44, ranked second in mortality of common cancers in Vietnamese women in 2002 (16). Previous studies in Vietnam estimated the overall 5-year survival rate to be 85.1%; this rate varied with the stage of breast cancer. The prognostic factors, the hormonal receptors, such as estrogen receptor (ER), progesterone receptor (PR), and hormone therapy, were reported inconsistently in these studies (17, 18). While previous studies investigated survival time only for specific patient groups such as premenopausal breast cancers with/without positive hormone receptors or patient groups who received supportive treatment with chemotherapy and/or hormones, the present study used a more general sample and included demographic factors not previously considered in Vietnam. The objectives of the study were to estimate the survival probability at 1, 3, and 5 years, following diagnosis of breast cancer patients, and to determine the effects of age, ethnicity, stage at diagnosis, marital status, educational level, type of primary treatment, and hormone therapy on mortality of breast cancer in Vietnam. The results of the study could help health policy makers engaged in planning for breast cancer interventions and can be used in models of cost-effectiveness analysis of interventions for breast cancer in the country.

**Policy recommendations from the study**

- An early detection strategy for breast cancer should be developed to improve life expectancy of women with breast cancer in Vietnam.
- Breast cancer education and awareness should be supported hand in hand with early detection programs.
- Financial support policies should be considered to promote access of women to appropriate diagnosis and treatment, particularly for the most economically disadvantaged.

## Methods

### Study settings

The study was conducted in Ho Chi Minh City and Thua Thien Hue province. Ho Chi Minh City in the South of Vietnam is the largest economic center in the country and the most crowed city, with a population of 7,196,100 in 2009 (19). The Ho Chi Minh Cancer Registry, a population-based cancer registry established in 1990, is one of the two cancer registries in Vietnam recognized by the International Association of Cancer Registries (IACR) (20, 21). Thua Thien Hue province is located in central Vietnam; its capital, Hue City, is famous as a cultural center of the country. The province had 1,087,600 people in 2009 (19). Hue Central Hospital is responsible for a cancer registry covering all provinces in the central region. It was established in 2000 and developed into a population-based cancer registry in 2008. These cancer registries do not include data on follow-up or death certificates of cancer patients (20). Breast cancer is the most common cancer among women in both study settings with the age standardized rate (ASR) of the disease estimated at 21/100,000 in Ho Chi Minh City and 11.9/100,000 in Thua Thien Hue province between 2004 and 2008 (22)

### Data source and subjects

The morbidity data of the study came from the Ho Chi Minh Cancer Registry and the medical record reviews from Hue Central Hospital.

Inclusion criteria: all cases of female breast cancer with code of C 50 (ICD-10 version) (23) who met the following criteria were included in the study: age between 35 and 75 years; diagnosis with primary breast cancer in the period 2001–2006; resident of Ho Chi Minh City or Thua Thien Hue province; staging at diagnosis based on tumor/nodes/metastasis staging system (TNM) of the Union for International Cancer Control (UICC) (24).

A total of 1,584 cases of breast cancer were eligible for the study. This included 1,427 cases registered between 2003 and 2006 in Ho Chi Minh City. The remaining 157 cases were diagnosed between 2001 and 2006 in Thua Thien Hue province. Secondary data from the Cancer Registry in Ho Chi Minh City, and medical records in Hue Central Hospital provided information on age, ethnic group, date of diagnosis, stage at diagnosis, and the primary treatment following diagnosis. Those patients would be on the track in their community until December 31, 2010. The result of follow-up left 948 patients or their relatives (if the patients were deceased) available for direct interview. There was incomplete follow-up data for 636 patients (40.2%). The most common reason for incomplete follow-up was migration, particularly in Ho Chi Minh City. Population shifts are understandable in the biggest and the most crowded city in Vietnam. The data in Table 1 compare the characteristics between the two groups of patients, with and without loss to follow-up.

**Table 1:** Characteristics of breast cancer patients for groups with and without loss of follow-up

Characteristics	Group without loss of follow-up (*N=*948),%	Group with loss of follow-up (*N=*636),%	*p* *
Ethnic group			
Majority (Kinh)	94.41	95.75	0.231
Minority	5.59	4.25	
Age group			
<40	14.24	13.99
40–49	43.25	36.62
50–59	26.05	29.72	0.436
60–69	13.71	13.21	
≥70	2.74	3.46	
Stage at diagnosis			
Stage 0	0.42	0.94	
Stage I	10.76	11.01	0.094
Stage II	61.18	57.08	
Stage III	19.41	24.21	
Stage IV	8.23	6.76	
The primary treatment			
Surgery	78.69	72.96
Radiation	0.42	0.94	0.039
Chemotherapy	10.55	11.32	
Hormone therapy	0.11	0.31	
No treatment	10.23	14.47	

* Pearson chi-square.

There was no statistically significant difference in most characteristics of breast cancer patients between these two groups (*p*>0.05). The exception was the primary treatment received. However, this difference was small (*p*=0.039). The group lost to follow-up was excluded from the survival analysis in the study.

Follow-up information was obtained from direct interviews of 948 patients or their relatives, using structured questionnaires. Information on marital status, educational level, hormone therapy during 5 years, patient’s status (alive or dead), date of death, and causes of death were collected for each patient.

### Prognostic factors

We examined the effects of ethnic group, age, marital status, educational level, stage at diagnosis, type of primary treatment and hormone therapy on survival time of breast cancer patients.

Ethnicity in the sample was classified as majority (Kinh) or minority (primarily Chinese). The age variables were stratified into five groups: aged <40; 40–49; 50–59; 60–69; ≥70. Marital status was recorded as married or unmarried. Unmarried women included those who had never married before they were diagnosed with breast cancer. Educational level was categorized into five groups: illiterate; primary school; secondary school; high school and junior college, or higher. Stage at diagnosis (clinical or pathological) was analyzed as stage 0 (in situ); stage I, stage II (including stage IIA and stage IIB), stage III (including stage IIIA and stage IIIB), and stage IV. Because so few patients presented with stage 0, stage I and stage 0 were combined for the analysis. Primary treatment was classified into one of five categories based on the initial treatment received following diagnosis. The treatment categories were surgery, radiotherapy, chemotherapy, hormone therapy, and no treatment (treatment was not received or was unknown). Patients or their relatives’ recall of prescriptions for Tamoxifen or similar medication during the 5 years after primary treatment was used as an indicator of hormone treatment in this study.

### Statistical analysis

The survival duration of each case was determined as the difference in time (months) between the date of initial diagnosis until the date of death (by breast cancer only), or the closing date of follow-up. Death by other causes was considered censored results in the study. Potential follow-up time ranged from 4 to 10 years. The overall survival of patients with breast cancer at 1, 3, and 5 years, following diagnosis was calculated by the Kaplan-Meier method. A Cox regression model was used to examine the effects of characteristics of patients and treatments on survival time. The following variables were included in the model as predictors: ethnic group, age group, marital status, education level, stage at diagnosis, primary treatment, and hormone therapy. A goodness-of-fit (GOF) test was conducted to assess the proportional hazard (PH) assumptions of the Cox model for given predictor variables (Table 2). The findings indicated that variable education level and variable stage at diagnosis did not satisfy PH assumptions (*p*<0.05). We therefore fitted the extended Cox model in which these variables were analyzed as time-dependent variables (25).

**Table 2:** Goodness-of-fit test assessing proportional hazards assumption

Predictors	rho*	Chi-square test	df**	*p*
Ethnic group	−0.06846	1.28	1	0.2571
Age group	0.03237	0.29	1	0.5871
Marital status	−0.02643	0.19	1	0.6619
Education level	−0.16793	7.48	1	0.0062
Stage at diagnosis	−0.33341	27.11	1	0.0000
Primary treatment	0.09538	2.76	1	0.0967
Hormone therapy	0.11012	3.12	1	0.5871
Global test		38.11	7	0.0000

* The correlation coefficient between the residuals and time.

** Degree of freedom.

In addition, the possible interaction effects of education level, marital status, hormone therapy, and primary treatments with stage at diagnosis were considered in this full model.

The formula for the extended Cox model for time-dependent variables was applied as following:

$$\text{h}(\text{t},{\text{X}}_{\text{i}},{\text{X}}_{\text{j}}(\text{t}))={\text{h}}_{\text{o}}(\text{t})\text{exp}\left[\sum_{i=1}^{p1}\beta \text{iXi}+\sum_{j=1}^{p2}\delta \text{jXj}(\text{t})\right]$$

where

h(t, X_i_, X_j_(t)) is Hazard function—expresses the hazard at time *t* for an individual with a given specification of set of time-independent predictor variables and time-dependent predictor variables.

ho(t) is Baseline hazard function.

X_i_ denotes the *i*th time-independent predictor variable.

X_j_(t) denotes *j*th time-dependent predictor variable.

p_1_X_i_ is the entire collection of time-independent predictors (including variables, namely ethnic, married, treatment, hormone, age in the study).

p_2_X_j_(t) is the entire collection of time-dependent predictors at time t (including variables stage(t), education(t), treatment*stage(t), married*stage(t), education*stage(t), hormone*stage(t) in the study).

ß_i_: regression coefficient for time-independent predictor variables.

δ_j_: regression coefficient for time-dependent predictor variables.

Model assumes that hazard at the time t depends on the value of X_j_ (t) at the same time.

Then hazard ratio (HR) formula for extended Cox model is:

$$\text{HR}(\text{t})=\text{exp}\left[\sum_{\text{i}=1}^{\text{p}1}\beta [{\text{X}}_{\text{i}}^{*}-{\text{X}}_{\text{i}}]+\sum_{\text{i}=1}^{\text{p}2}\delta [{\text{X}}_{\text{j}}^{*}(\text{t})-{\text{X}}_{\text{j}}(\text{t})]\right]$$

This formula describes the ratio of hazards at a particular time t that compares two specifications of time-independent predictors (X^*^ and X) and those of time-dependent predictors at time t (X^*^(t) and X(t)). The coefficient δ represents the overall effect of the corresponding time-dependent variables, considering all times at which this variable has been measured in the study (25).

A reduced extended Cox model was then carried out by excluding interaction terms that were not statistically significant. In the final model, the prognostic factors that showed statistically significant effects were analyzed according to subgroups to determine the effect of each stratum on survival time for breast cancer patients. A *p*-value of less than 0.05 was considered statistically significant.

## Results

Study subject characteristics are reported in Table 3. The mean age of patients at the time of diagnosis was 50 years. The most frequent age group was that from 40 to 49 years (43% of the patients). Most the women were married (83.1%). The education level of the study population was low, with 30.1 and 27.8% of women having completed primary school and secondary school, compared with 26.4 and 11.5% of those having attended high school and higher levels. Among the women, 4.2% were illiterate. The majority of the women had been diagnosed with breast cancer at stage II at primary diagnosis (61.2%). Late stage diagnosis (stage III and stage IV) was also considerable, making up 19.4 and 8.2% of the study population, respectively. Surgery was the most common treatment for patients following diagnosis with breast cancer (78.7%). Significantly, 10.2% of all the patients did not receive any type of primary treatment and 10.6% of cases were treated with chemotherapy after diagnosis. There were 74.6% of cases who reported receiving hormone therapy during 5 years following the primary treatment.

**Table 3:** Characteristics of patients with breast cancer

Characteristics	Number of patients	Percent
Ethnic group		
Majority group	894	94.3
Minority group	54	5.7
Age at diagnosis	
Mean (SD)	50 (9.2)	
Age group	
<40	103	10.9
40–49	408	43.0
50–59	270	28.5
60–69	131	13.8
≥70	36	3.8
Marital status	
Married	788	83.1
Unmarried	160	16.9
Education level	
Illiterate	40	4.2
Primary school	285	30.1
Secondary school	264	27.8
High school	250	26.4
Higher	109	11.5
Stage at diagnosis	
Stage 0 and I	106	11.2
Stage II	580	61.2
Stage III	184	19.4
Stage IV	78	8.2
The first treatment	
Surgery	746	78.7
Radiotherapy	4	0.4
Chemotherapy	100	10.6
Hormone therapy	1	0.1
No treatment	97	10.2
Hormone therapy	
Yes	707	74.6
No	241	25.4
Total	948	100

**Fig. 1:**
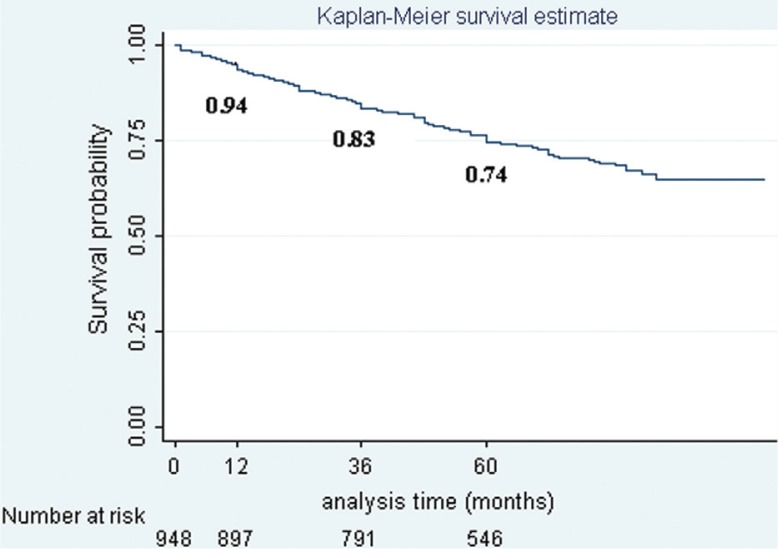
Overall survival rate at 1, 3, and 5 years following diagnosis for breast cancer.

The overall survival rate declined over time and was estimated to be 0.94, 0.83, and 0.74 at 1, 3, and 5 years, respectively, following diagnosis for breast cancer (Fig. 1).

**Fig. 2:**
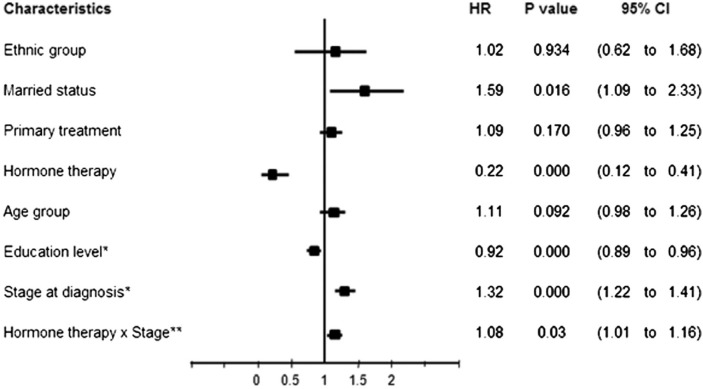
The extended Cox model assessing the effect of characteristics of patients on mortality from breast cancer.

The findings in Table 4 show evidence of interaction between hormone therapy and stage at diagnosis on the survival time (*p=*0.023), but there was no significant interaction effect between primary treatment, marital status, education level, and stage on risk of death for breast cancer cases in this study (*p*>0.05). Estimates of the regression coefficients for time-dependent variables indicate that the death hazard decreases with education level and increases with the stage at diagnosis (Table 4). The results from the extended Cox model analysis, excluding insignificant interaction terms, revealed four characteristics that had prognostic value for survival probability for breast cancer, including marriage (*p=*0.016), education level (*p*<0.001), stage at diagnosis (*p<*0.001), and hormone therapy (*p<*0.001) (Fig. 2). Marital status had the strongest effect on risk of death, with HR of 1.59 (95% CI: 1.09 to 2.33), followed by stage at diagnosis (HR: 1.32; 95% CI: 1.22 to 1.41). A later stage of breast cancer and the status of ‘married’ were related to poor prognosis for survival probability. In contrast, a higher education level and hormone therapy reduced mortality, with HR of 0.92 (95% CI: 0.89 to 0.96) and HR of 0.22 (95% CI: 0.12 to 0.41), respectively.

**Table 4:** Extended Cox model, including patient characteristics and interaction terms

	Hazard ratio	*p*	95% CI
Ethnic group	1.01	0.961	0.61–1.67
Marital status	2.41	0.093	0.86–6.74
Primary treatment	0.89	0.542	0.61–1.30
Hormone therapy	0.22	0.000	0.12–0.40
Age group	1.11	0.100	0.98–1.25
Education level*	0.86†	0.034	0.76–0.99
Stage at diagnosis*	1.25‡	0.019	1.04–1.51
Primary treatment×stage**	1.02	0.235	0.99–1.06
Marital status×stage**	0.95	0.367	0.85–1.06
Education level×stage**	1.03	0.325	0.98–1.08
Hormone therapy×stage**	1.08	0.023	1.01–1.16

95% CI: 95% confidence interval for hazard ratio.

* Variables continuously vary with respect to time.

** Interaction terms defined as the product of primary treatment, marital status, education level, hormone therapy, and stage at diagnosis

† Estimate of regression coefficient for variable education level is −0.14.

‡ Estimate of regression coefficient for variable stage at diagnosis is 0.22.

A further analysis of these prognostic factors is shown in Table 5. The hazard ratio for stage IV was 2.27 times higher than that for stage I. Married women faced nearly 60% higher risk of death than unmarried women. Women with an education level of high school and higher and women who had received hormone therapy reduced risk of death by approximately 10 and 80%, respectively, compared with those classified as illiterate and those without hormone therapy.

**Table 5:** Subgroup analysis of potential prognostic factors

Prognostic factors	Hazard ratio	p	95% CI
Marital status			
Unmarried	1 (baseline)		
Married	1.53	0.030	1.04–2.24
Hormone therapy			
No	1 (baseline)		
Yes	0.24	0.000	0.13–0.44
Education level*			
Illiterate	1 (baseline)		
Primary school	1.00	0.974	0.83–1.20
Secondary school	0.95	0.571	0.78–1.14
High school	0.79	0.020	0.64–0.96
Higher	0.79	0.046	0.62–0.99
Stage at diagnosis*
Stage I	1 (baseline)		
Stage II	1.24	0.060	0.99–1.56
Stage III	1.64	0.000	1.29–2.09
Stage IV	2.27	0.000	1.71–2.99
Hormone therapy×stage**	1.07	0.053	0.99–1.14

95% CI: 95% confidence interval for hazard ratio.

* Variables continuously vary with respect to time.

** Interaction terms defined as the product of hormone therapy and stage at diagnosis.

## Discussion

The study showed similarities in the epidemiology of breast cancer in Vietnam and other Asian countries. The disease was most common in women aged between 40 and 49, while in the West incidence peaks among women aged between 60 and 70 years (1–3). The majority of women in the study population were newly diagnosed at stage II of breast cancer, whereas 60–70% of cases in developed countries were detected at earlier stages (stage I and stage 0) (1, 2). Consequently, surgery was the most common primary treatment in the study. Mastectomy or conserving surgery and/or axillary dissection are the favored treatments for breast cancer patients at early stages (stage I and stage II) in almost all countries (2). The survival probability of breast cancer in this study population decreased with time from initial diagnosis. Overall survival rates at 1, 3, and 5 years in our study were higher than those in a similar study from Chennai (known as previous Madras), India, in 1997, at 94; 83, and 74% versus 80, 58, and 48%, respectively (6). The 5-year overall survival of the study population was also higher than that of Uganda (56%) in 2008 (5). However, the rate revealed in the study was rather lower than those in other Asian countries, such as China (76.5%), Taiwan (78.37%), Japan (76.1–86.1%), and South Korea (83.5%) (2, 7), and even lower than previous results in North Vietnam (85.1%) (17, 18). This figure is still lower when compared with Western countries such as Sweden (89%), Canada (86%), and the United States (88%) (2).

Among prognostic factors, those who were diagnosed at a higher stage demonstrated a poor prognosis for survival time. This result is consistent with previous findings in many studies all over the world (4–7, 26). One reason for the lower survival rate in our study is the late stage at which breast cancer is detected compared to developed countries (2). Moreover, the absence of primary treatment for 10.2% of the study population compared with previous studies in Vietnam, in which all patients received standard treatment after diagnosis, may explain the poorer survival rate of breast cancer patients in this study. Lack of appropriate treatment could also be the reason for the higher mortality in our study compared to other Asian countries with the same stage distribution at diagnosis (3–5, 26). Indeed, although there have been many efforts in cancer control in Vietnam, these activities have only met 15% of demand. The country is facing a lack of infrastructure, equipment, and human resources in oncology. Programs for the early detection of breast cancer have not yet been implemented nationwide. Cancer awareness in the community is still low. These are the main reasons for presentation at hospital with late stages of cancer. In addition, most facilities with capacity to treat cancer are located in a few big cities; hence, most cancer patients must be referred to the central level (27). Our findings suggest that early detection of breast cancer along with the availability and accessibility of appropriate treatment should be recommended to improve life expectancy for women with the disease in Vietnam.

Being married appeared as the most unfavorable prognostic factor for survival probability for women with breast cancer in our study. A review by Falagas et al. (2007) showed that the effect of marital status on breast cancer outcome varied across studies (9). The benefits of emotional support, good lifestyle, and stable economics were likely protective factors on survival for married women with cancer in the previous studies (6, 9, 10, 28). However, other recent studies indicated that social support and social network had a more important role in reduced mortality for breast cancer, and that support received from social networks improved survival in unmarried women (29, 30). The marital status alone had no significant influence on longer survival of women with breast cancer (31–33). In the context of Vietnam, married women, especially women with many children, often faced difficult economic circumstances. The financial burden of the treatment course for breast cancer could be a barrier to seeking care and to appropriate treatment compliance, more importantly, when patients must pay out of their pockets for health care services. This may contribute to the higher mortality of breast cancer among married women in this study. In this situation, universal coverage of health insurance should be promoted in Vietnam. Besides, the government budget to support vulnerable groups in health care expenditure should include cancer patients for whom the cost exceeds their ability to pay. This combination would encourage the patients in compliance with their long-term treatment and thus contribute to reducing deaths from cancers, including breast cancer.

The association between education level and survival rate of breast cancer was inconsistent across studies around the world (6, 12, 34). Generally, our study found a positive effect of education level on survival for breast cancer patients. The higher the level of education, the lower the risk of death due to breast cancer. This finding may reflect increasing knowledge and awareness of the disease; appropriate accessibility to health services; and good compliance to treatment and clinical follow-up by the higher education groups (6, 12). As mentioned earlier, the limited awareness and knowledge of cancer at the community level has been common in Vietnam (27). It is necessary to educate the public on breast health. The communication should be relevant to all subjects for the purposes of early detection of breast cancer, prevention of breast cancer, and prevention of death from the disease.

The final prognostic factor found in the study was hormone therapy within a 5-year treatment course. This therapy reduced the risk of death among breast cancer patients in the study population, in accordance with previous reports (35, 36). The effect of hormone therapy on survival improvement was often mentioned along with the influence of hormone receptors. An overview of randomized trials indicated that Tamoxifen was more effective in ER-positive than in ER-negative women (36). More importantly, Crowe et al. concluded that the presence of these hormonal factors impacted on longer survival for breast cancers (37). Prior studies in Vietnam found inconsistent effects of hormone receptors on prolonging survival time of women with breast cancer. However, all patients in these studies were taking hormone therapy during their treatment course (17, 18). It is difficult to identify the role of hormone therapy or hormone receptors as independent prognostic factors. A study by Caldarola et al. reported no significant relationship between ER or PR and improved survival (38). In our study, some of the patients had unknown hormonal receptors because the assay techniques used for ER/PR testing were not available in Hue Central Hospital before 2006. They were prescribed hormone therapy based on the experience of specialized doctors (personal communication, Dr. Nguyen Dinh Tung, Oncology Department, Hue Central Hospital). The greatest improvement in survival from breast cancer of hormonal therapy in our study (HR: 0.22) was strong enough to confirm this as an independent prognostic factor. Moreover, the presence of hormone therapy reduced the effect of stage on the risk of death from breast cancer (HR of interaction term: 1.08).

A number of limitations should be considered when interpreting the findings of this study. The high proportion of patients lost to follow-up (40.2%) and their exclusion from the survival analysis may have resulted in selection bias although their characteristics did not differ from those included in the analysis. The data were collected over the period 2001–2006 and do not reflect current utilization of advanced treatment methods and new medications for breast cancer treatment, which could affect the opportunity to improve survival probability in the study population. Moreover, diagnosis of breast cancer in the study population was defined as the time when patients presented at hospitals for primary treatment, so the lead-time bias could be a potential problem. The risk of recall bias can occur with information on education level and hormone therapy following primary treatment. Although the study population was representative for two big cities in Vietnam with different characteristics in socioeconomics and epidemiology of breast cancer, the generalizability of the study findings should be considered. The presence of high level hospitals might increase accessibility and availability of relevant treatment; the survival time estimates of our study could be better than those in other areas of the country where diagnosis and treatment techniques varied (15, 22). Despite these limitations, this is the first population-based survival analysis for breast cancer in Vietnam. Risk factors on mortality of breast cancer were identified. The results will contribute important information to cost-effectiveness analysis of interventions for breast cancer and will help health policy makers engaged in planning for programs to reduce breast cancer mortality. Among the risk factors, stage at diagnosis showed a strong relation to mortality on breast cancer. Developing early detection strategies for breast cancer to shift the stage at presentation to more favorable early stages is necessary to improve the life expectancy of women with breast cancer in Vietnam. Breast cancer education and awareness should be supported hand-in-hand with early detection programs. The poorest prognosis on survival time of married women with breast cancer suggested that financial support policies are needed to promote access of women, especially poor women, to appropriate diagnosis and treatment. Besides, the most important factor to achieve the goal of reducing breast cancer mortality is that infrastructure, equipment, and human resources for diagnosis and breast cancer treatment should be made available, accessible, and affordable to women in whom early detection strategies identify breast cancer. Overall, these recommendations are drawn from the evidence found in the study. Decisions about these policies should consider the available resources in the country and should also inform directions for future plans.

## Conclusion

The survival probability of breast cancer patients in Vietnam decreased over time following diagnosis. Five-year survival rate was lower than that in other countries with similar distributions of stage at diagnosis. Married status, late stage at diagnosis, low education level, and lack of hormone therapy were prognostic factors for higher mortality of women with breast cancer. An early detection program with support policies could reduce breast cancer mortality in Vietnam.

## Ethical approval

Ethical approval for the data collection was obtained from the University of Khon Kaen, Thailand (where the study was designed as part of a doctoral study program). In addition, approval for implementing the study at the sites was obtained from Health Services of Thua Thien Hue province and Ho Chi Minh City.

## References

[1] Peter B, Bernard L World cancer report 2008 [pdfs online].

[2] Leong SP, Shen ZZ, Liu TJ, Agarwal G, Tajima T, Paik NS (2010). Is breast cancer the same disease in Asian and Western countries?. World J Surg.

[3] Bray F, McCarron P, Parkin DM (2004). The changing global patterns of female breast cancer incidence and mortality. Breast Cancer Res.

[4] Meng L, Maskarinec G, Wilkens L (1997). Ethnic differences and factors related to breast cancer survival in Hawaii. Int J Epidemiol.

[5] Gakwaya A, Kigula-Mugambe JB, Kavuma A, Luwaga A, Fualal J, Jombwe J (2008). Cancer of the breast: 5-year survival in a tertiary hospital in Uganda. Br J Cancer.

[6] Gajalakshmi CK, Shanta V, Swaminathan R, Sankaranarayanan R, Black RJ (1997). A population-based survival study on female breast cancer in Madras, India. Br J Cancer.

[7] Lee JH, Yim SH, Won YJ, Jung KW, Son BH, Lee HD (2007). Population-based breast cancer statistics in Korea during 1993–2002: incidence, mortality, and survival. J Korean Med Sci.

[8] Anderson BO, Yip CH, Smith RA, Shyyan R, Sener SF, Eniu A (2008). Guideline implementation for breast healthcare in low-income and middle-income countries: overview of the Breast health global initiative global summit 2007. Cancer.

[9] Falagas ME, Zarkadoulia EA, Ioannidou EN, Peppas G, Christodoulou C, Rafailidis PI (2007). The effect of psychosocial factors on breast cancer outcome: a systematic review. Breast Cancer Res.

[10] Kravdal H, Syse A (2011). Changes over time in the effect of marital status on cancer survival. BMC Public Health.

[11] Heck KE, Wagener DK, Schatzkin A, Devesa SS, Breen N (1997). Socioeconomic status and breast cancer mortality, 1989 through 1993: an analysis of education data from death certificates. Am J Public Health.

[12] Hussain SK, Altieri A, Sundquist J, Hemminki K (2008). Influence of education level on breast cancer risk and survival in Sweden between 1990 and 2004. Int J Cancer.

[13] Dunnwald LK, Rossing MA, Li CI (2007). Hormone receptor status, tumor characteristics, and prognosis: a prospective cohort of breast cancer patients. Breast Cancer Res.

[14] Fletcher AS, Erbas B, Kavanagh AM, Hart S, Rodger A, Gertig DM (2005). Use of hormone replacement therapy (HRT) and survival following breast cancer diagnosis. Breast.

[15] Duc NB (2010). Epidemiology and program of control and prevention for cancer: preliminary report of national cancer project period 2008–2010. Viet J Oncol.

[16] (2008). The Burden of Cancer in Asia. Pfizer facts.

[17] Dieu B, Trang NTP, Binh ND (2010). Premenopausal breast cancers with positive hormonal receptors: 5 year survival rate of surgical department of National Cancer hospital—Tam Hiep campus. Viet J Oncol.

[18] Thang VH, Tu DA, Hoa NTT, Toan VQ, Hung TT, Huong TTTH (2010). Analysis of predictive value of epidermal growth factors in breast cancer. Viet J Oncol.

[19] General Statistics Office Population of each region in Vietnam in 2010.

[20] Nguyen Dinh Tung (2010). Cancer incidence in the population of Thua Thien Hue province, Vietnam, 2001–2009. J Sci, Hue University.

[21] International Association of Cancer Registries IACR Membership list. http://www.iacr.com.fr/asiact.htm.

[22] Duc NB, Thuan TV, Can DT, Dieu B, Nga NTH, Thang ND (2009). Situation of female breast cancer in some provinces and cities period 2001–2007. Viet J Oncol.

[23] World Health Organization (2010). International classification of diseases 10th revision. ICD-10 version.

[24] Blogspot.com TNM classification help: Breast tumours. http://cancerstaging.blogspot.com/2005/02/breast-tumours.html.

[25] David GK, Mitchel K (2005). Survival analysis. A self-learning text.

[26] Jack RH, Davies EA, Moller H (2009). Breast cancer incidence, stage, treatment and survival in ethnic groups in South East England. Br J Cancer.

[27] Vietnamscope http://www.vietnamscope.org/index.php?option=com_content&view=article&id=99&Itemid=139.

[28] Osborne C, Ostir GV, Du X, Peek MK, Goodwin JS (2005). The influence of marital status on the stage at diagnosis, treatment, and survival of older women with breast cancer [abstract]. Breast Cancer Res Treat.

[29] Kroenke CH, Kubzansky LD, Schernhammer ES, Holmes MD, Kawachi I (2006). Social networks, social support, and survival after breast cancer diagnosis. J Clin Oncol.

[30] Antonucci TC (1987). An examination of sex differences in social support among older men and women. Sex Roles.

[31] Laino C (2005). Marital status doesn't affect breast cancer outcomes. Oncol Times.

[32] Goodwin JS, Hunt WC, Key CR, Samet JM (1987). The effect of marital status on stage, treatment, and survival of cancer patients. JAMA.

[33] Goodwin PJ, Leszcz M, Ennis M, Koopmans J, Vincent L, Guther H (2001). The effect of group psychosocial support on survival in metastatic breast cancer. N Engl J Med.

[34] Valanis B, Wirman J, Hertzberg VS (1987). Social and biological factors in relation to survival among black vs. white women with breast cancer. Breast Cancer Res Treat.

[35] Sener SF, Winchester DJ, Winchester DP, Du H, Barrera E, Bilimoria M (2009). The effects of hormone replacement therapy on postmenopausal breast cancer biology and survival. Am J Surg.

[36] Early Breast Cancer Trialists’ Collaborative Group (EBCTCG) (2005). Effects of chemotherapy and hormonal therapy for early breast cancer on recurrence and 15-year survival: an overview of randomised trials. Lancet.

[37] Crowe JP, Gordon NH, Hubay CA, Shenk RR, Zollinger RM, Brumberg DJ (1991). Estrogen receptor determination and long term survival of patients with carcinoma of the breast. Surg Gynecol Obstet.

[38] Caldarola L, Volterrani P, Caldarola B, Lai M, Jayme A, Gaglia P (1986). The influence of hormone receptors and hormonal adjuvant therapy on disease-free survival in breast cancer: a multifactorial analysis. Eur J Cancer Clin Oncol.

